# Application of the Nicoya OpenSPR to Studies of Biomolecular Binding: A Review of the Literature from 2016 to 2022

**DOI:** 10.3390/s23104831

**Published:** 2023-05-17

**Authors:** Eliza K. Hanson, Rebecca J. Whelan

**Affiliations:** Department of Chemistry, University of Kansas, Lawrence, KS 66045, USA; e.hanson@ku.edu

**Keywords:** localized surface plasmon resonance, Nicoya Life Sciences, OpenSPR, binding affinity

## Abstract

The Nicoya OpenSPR is a benchtop surface plasmon resonance (SPR) instrument. As with other optical biosensor instruments, it is suitable for the label-free interaction analysis of a diverse set of biomolecules, including proteins, peptides, antibodies, nucleic acids, lipids, viruses, and hormones/cytokines. Supported assays include affinity/kinetics characterization, concentration analysis, yes/no assessment of binding, competition studies, and epitope mapping. OpenSPR exploits localized SPR detection in a benchtop platform and can be connected with an autosampler (XT) to perform automated analysis over an extended time period. In this review article, we provide a comprehensive survey of the 200 peer-reviewed papers published between 2016 and 2022 that use the OpenSPR platform. We highlight the range of biomolecular analytes and interactions that have been investigated using the platform, provide an overview on the most common applications for the instrument, and point out some representative research that highlights the flexibility and utility of the instrument.

## 1. Introduction

Surface Plasmon Resonance (SPR) is a popular technique to measure binding affinity and kinetics of biomolecular analytes of interest because it is label-free, in real time, sensitive, and broadly applicable. The first report of SPR for biosensing was in 1983 [1,2], and it has become a widely used technique in the forty years since then. The analytes it can measure span a broad range including small molecules [3,4], nucleic acids [5,6], proteins [7,8], antibodies [9], and cells [10]. It is a versatile technique and has also been used for epitope mapping [11,12], quantitative detection of biomarkers [13,14], and inhibition studies [9,15].

**Figure 1:**
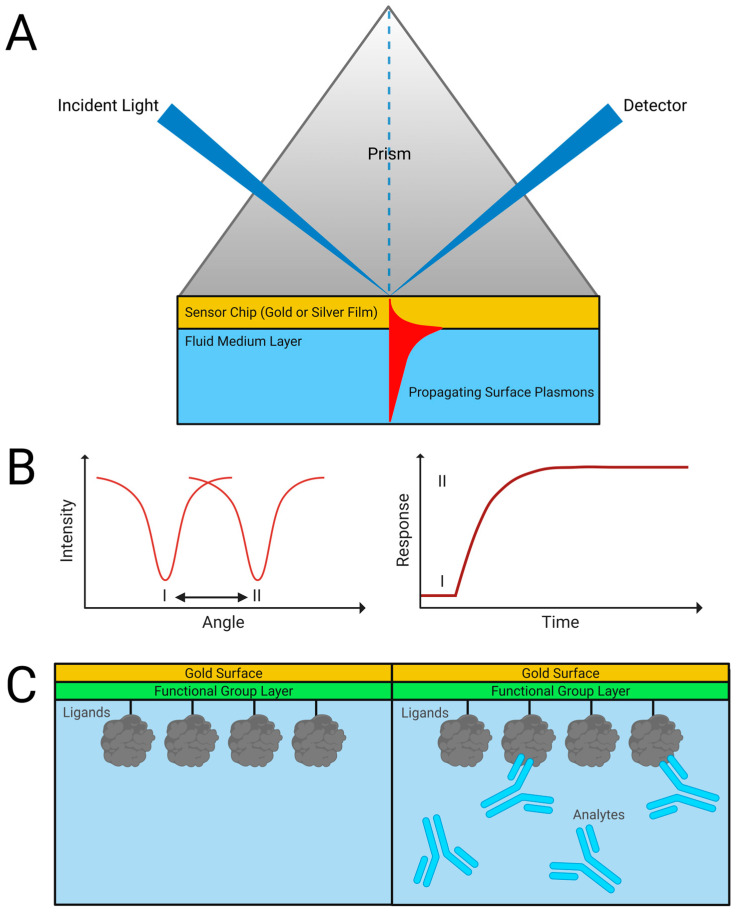
Traditional SPR technique. (**A**) Kretschmann geometry for an SPR sensor allows for the measurement of (**B**) light absorbance during refractive index changes caused when (**C**) binding analytes interact with immobilized ligands on the surface. Adapted from [20].

The phenomenon of surface plasmon resonance enables the technique. The energy of incident photons can be coupled with electrons in the sensor surface at a precise angle of incidence. The charge density wave that occurs is described as surface plasmons, and this plasmon oscillation is responsible for the electric field generated on the sensor surface, most typically a noble metal coating [2,16,17]. Many commercial SPR instruments use the attenuated total reflection method, employing a high-refractive index prism set up in the Kretschmann geometry (Figure 1). The angle of incidence responsible for resonance is determined by the refractive index of the sensor and is influenced by the minor changes that occur when ligands are immobilized on the sensor or when analytes bind [17,18]. This relationship is extremely sensitive, and binding on the surface can be quantified by measuring changes in the reflected light [17]. In the traditional configuration, the wavelength of excitation light is much smaller than the dimensions of the metallic film on the sensing surface, enabling the propagation of plasmons through the surface [19].

**Figure 2:**
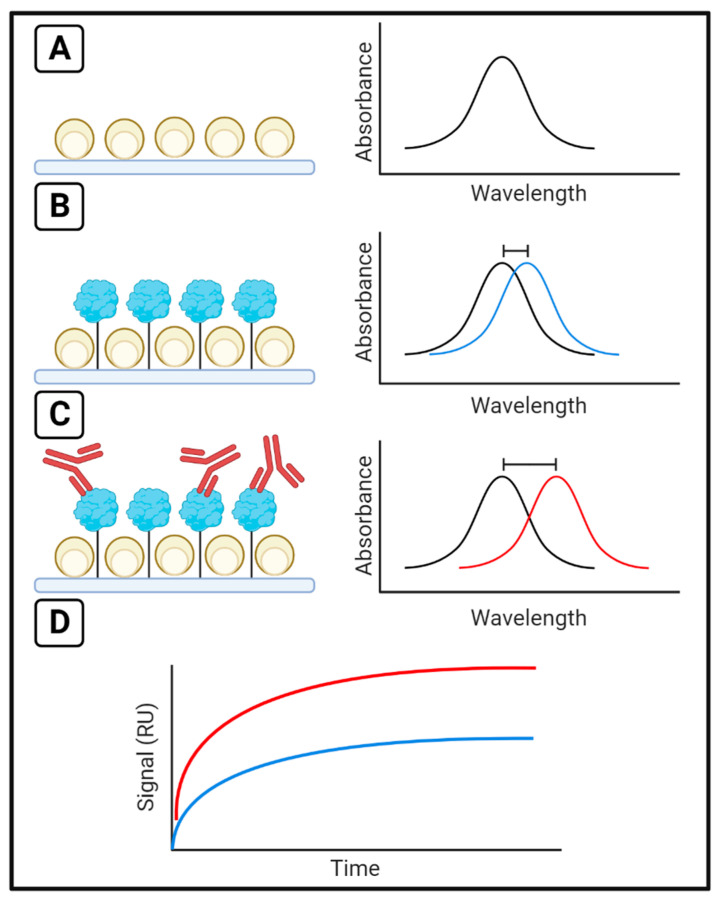
The LSPR Technique. (**A**) Maximum absorbance occurs at a given resonance wavelength for a sensor surface. This wavelength shifts (**B**) when ligands are immobilized or (**C**) analytes are injected, and by monitoring the (**D**) change in resonance wavelength over time, binding can be measured in response units (RU). The blue line represents signal change upon ligand binding, as in (**B**), and the red line represents signal change upon analyte binding, as in (**C**).

When the size of the nanoparticles comprising the sensor surface is equal to or smaller than the wavelength of incident light, the free electrons have a collective oscillation that is termed localized surface plasmons [21,22]. The collective oscillation has a maximum absorbance at the resonance wavelength. By monitoring the change in the resonance wavelength, binding at the surface can be quantified (Figure 2). This variation of the method is called Localized Surface Plasmon Resonance (LSPR) [19,21]. The small size of the nanoparticles on the sensing surface limits the depth of the sensing region, limiting background noise on the binding measurement. The sensing surface is customizable owing to the use of nanoparticles, and parameters such as nanoparticle shape and composition allow for optimization of the sensing surface for greater sensitivity and resolution compared to SPR [19].

LSPR can be accomplished using affordable bench-top instrumentation, one example of which is the Nicoya OpenSPR (Nicoya Life Sciences, Waterloo, ON, Canada). The instrument has been used with a broad range of analytes including small molecules [23,24,25,26], peptides [27,28,29,30], proteins [27,28,29,31,32,33], nucleic acids [34,35,36,37], carbohydrates [38,39,40], and lipids [41,42]. Nicoya offers a wide selection of surface chemistries to be used with the OpenSPR instrument, ranging from the most popular carboxyl immobilization to more specific applications, such as liposome sensors.

Researchers have used this technique to assess the binding affinity and kinetics of potential new therapeutics and affinity reagents, to study aggregation, to perform competition studies or inhibition assays, and other applications. OpenSPR has been leveraged to study diverse biochemical questions, including critical problems in human health such as Alzheimer’s disease (AD) [43,44,45,46], non-small cell lung cancer (NSCLC) [47,48,49,50,51], and COVID-19 [34,52,53,54,55,56,57,58,59,60,61,62,63,64,65,66,67,68,69,70,71]. This paper provides the first comprehensive review of the literature reporting the use of the OpenSPR. 200 such publications have appeared in the decade since Nicoya’s founding.

## 2. Search Parameters

The search for papers citing the use of a Nicoya OpenSPR instrument was intended to be as comprehensive as possible. The papers were found first from the list referenced on Nicoya’s website under the Publications Table. This list was supplemented with papers found with the search terms “OpenSPR” and “Nicoya” on Web of Science, Google Scholar, and PubMed with publication dates between 2012 and 2022. While Nicoya was founded in 2012, the first papers citing the use of the OpenSPR instrument were published in 2016 [27,72,73]. Because this survey is intended to focus on peer-reviewed research articles, we excluded conference papers, thesis publications, preprints, and review articles. However, the sources in review articles found in the search were checked, and any papers citing the use of OpenSPR found through those means were also included in the collection. Finally, only papers using the OpenSPR instrument were included; Nicoya’s Alto instrument is beyond the scope of this paper. The above search uncovered 200 papers citing the use of the OpenSPR between 2016 and 2022. Though only a handful of papers appear in the first few years, the number of citations has risen quickly. The distribution of papers by year is given in Table 1.

## 3. The Nicoya OpenSPR Compared to Similar Commercial SPR Systems

The Nicoya OpenSPR was designed to be affordable and easy to use to make the technique more accessible. In addition to examining how the OpenSPR has been used by surveying the research work it has been used for, it is helpful to compare the instrument to other commercially available options in order to understand how the instrument compares (see Table 2).

The OpenSPR can be used to measure the same affinity range for binding partners of interest as the other three systems, and the range of association and dissociation rates it can measure is comparable. The OpenSPR uses a fixed volume to inject, while the Biacore X100 and the Sierra SPR-32 Pro both have a range of possible injection volumes, and the Alto only requires 2 μL of sample and uses digital microfluidics rather than millifluidics and a sample injection through a flow cell to manipulate samples. The OpenSPR has the widest range of possible flow rates and assay temperatures. With the addition of the XT autosampler, the OpenSPR has a comparable unattended run time.

Qiang and coworkers directly compared the Nicoya OpenSPR (which uses localized SPR) with a Biacore SPR (using the traditional reflected angle SPR detection) to investigate the anti-inflammatory activity of the dermcidin peptide binding to the epidermal growth factor receptor (EGFR) protein and found comparable results between the two instruments [80].

## 4. Binding Affinity and Kinetics

### 4.1. Principles of a Binding Assay

SPR is a powerful tool for real-time, label-free measurement of binding affinity and kinetics, and the vast majority of papers citing the use of the OpenSPR fit neatly underneath this umbrella [3,5,7,9,15,23,24,25,26,27,28,29,30,31,32,33,34,35,36,37,38,39,40,41,42,43,44,46,47,48,49,50,51,52,53,54,55,56,57,58,59,60,61,62,63,64,65,66,67,68,69,70,71,73,80,81,82,83,84,85,86,87,88,89,90,91,92,93,94,95,96,97,98,99,100,101,102,103,104,105,106,107,108,109,110,111,112,113,114,115,116,117,118,119,120,121,122,123,124,125,126,127,128,129,130,131,132,133,134,135,136,137,138,139,140,141,142,143,144,145,146,147,148,149,150,151,152,153,154,155,156,157,158,159,160,161,162,163,164,165,166,167,168,169,170,171,172,173,174,175,176,177,178,179,180,181,182,183,184,185,186,187,188,189,190,191,192,193,194,195,196,197,198,199,200,201,202,203,204,205,206,207,208].

**Figure 3:**
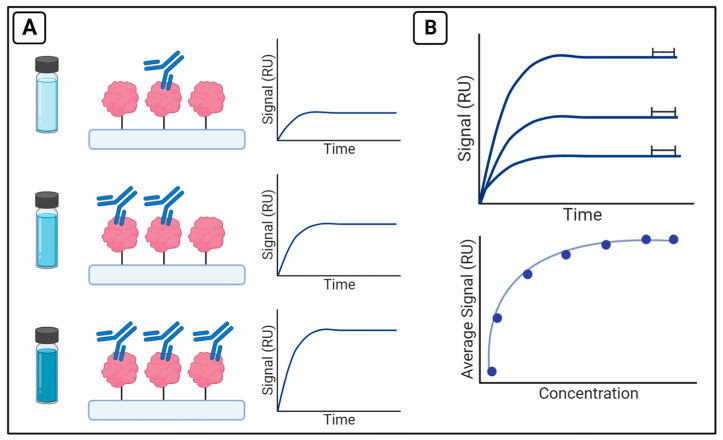
Experimental design for binding affinity and kinetics assays (**A**) After ligand immobilization, a range of analyte concentrations are injected over the surface of the chip and signal is recorded in RU. Following the experiment, the collected traces from each injection can be plotted. (**B**) Average signal from the end of each run (upper panel) can be plotted versus the concentrations to generate an isotherm and calculate K_D_ (lower panel) or fit directly by analysis software to calculate kinetic parameters such as k_on_ and k_off_.

A typical binding affinity and kinetics assay on the OpenSPR involves the immobilization of one binding partner of interest (the ligand), and the injection of another binding partner (analyte) over the surface. An analyte at various concentrations is injected over the immobilized ligand and the signal is measured in response units (RU). The signal corresponds to the shift in resonance wavelength as binding occurs on the surface and is plotted versus time. The collected traces from each injection of analyte are collected and analyzed to calculate affinity or the kinetic parameters of binding (Figure 3).

Nicoya offers a variety of sensor chips to immobilize ligands of interest with different surface chemistries. Some methods are permanent covalent immobilizations, such as the carboxyl chip, while others use affinity interactions, such as streptavidin-biotin or 6xHis-Ni-NTA. The types of sensors chosen by users of the OpenSPR are listed in Table 3. The work by Cathcart et al. has been omitted, as their work focuses on developing new types of sensor nanoparticles and does not utilize the commercially available chips [72,209,210]. While most investigations used one sensor type, a few papers cite using two different chips. In situations where two different sensors are available for the same type of coupling chemistry (such as carboxyl and amine sensors or streptavidin and biotin sensors), they have been combined into one category for clarity. The gold sensor chips were used both for thiol chemistry and for groups that performed their own functionalization on the surface.

**Figure 4:**
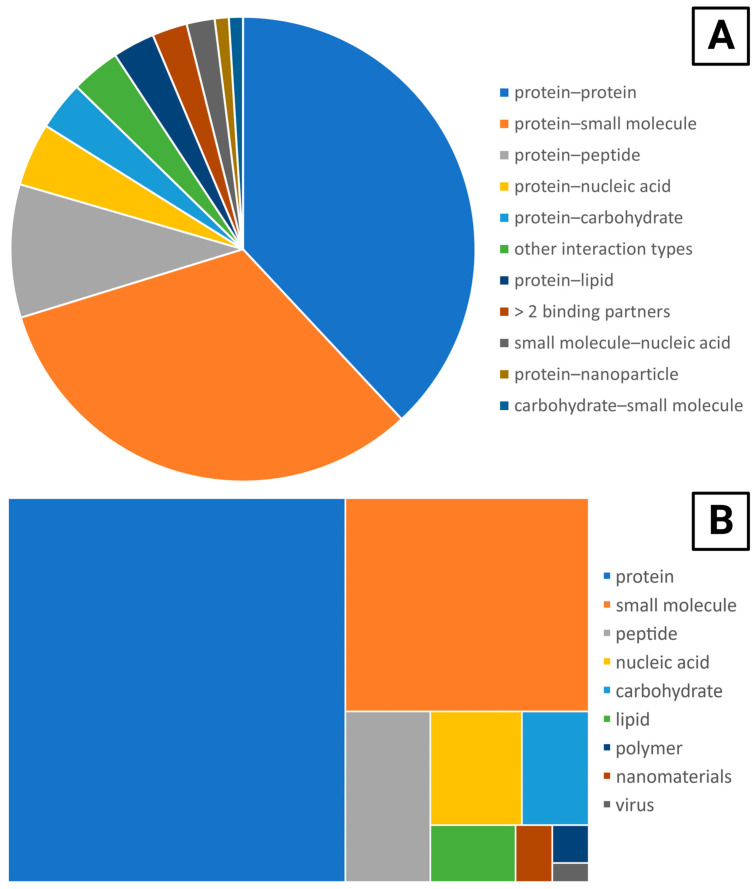
Applications of the OpenSPR to different kinds of molecular interactions and analytical targets. (**A**) The number of papers investigating each type of binding system, with several papers investigating more than one category. The “other interaction types” category were pairs of ligand–analyte interactions that were only cited by one paper each and includes carbohydrate–carbohydrate, polymer–small molecule, peptide–lipid, protein–virus, carbohydrate–peptide, polymer–protein, and nanofiber–protein. Papers that were characterizing nanoparticles [72,209,210] or surface aggregation [213] were omitted as they were investigating something other than a binding interaction. (**B**) The number of papers that investigated each type of target.

SPR is a flexible technique that can be applied to a range of targets from small molecules to larger analytes such as viruses (Figure 4A). The most common interaction type investigated was protein–protein, followed closely by protein–small molecule. The majority of papers were interested in two binding partners, but several papers were characterizing more than two, such as the papers looking at collaborative protein–protein–nucleic acid binding [211,212].

Looking at the analytical targets investigated separately from the categories of interaction types offers further insight into trends in the application of the OpenSPR (Figure 4B). Most papers investigated proteins binding with a variety of different binding partners. With 187 papers, the set of papers looking at protein binding is more than twice the size of the next largest category. In contrast to the widely varied protein binding partners, most papers investigating binding to small molecules are in the same category, with 66 of them in protein–small molecule out of 75 total papers that investigate a small molecule target.

There are a wide variety of reasons investigators use SPR, and some of the most common applications are assessing the binding of a potential therapeutic to its target [33,37,44,53,55,56,57,58,59,60,61,62,65,66,67,80,84,169,176,179,181,207] or to measure the affinity of a potential inhibitor [23,25,46,86,89,98,105,114,115,123,133,143,148,158,159,171,182,186,187,204]. The proposed therapeutics were sometimes novel compounds, such as the tasine derivate compounds synthesized by Yang and coworkers to suppress HeLa cells [33] or the bioactive iridium metal-complex developed by Ji et al. to eliminate excess reactive oxygen species (ROS) induced by spinal cord injuries (SCI) [179]. Researchers also investigated repurposing existing drugs to address critical public health problems, such as the work by Ge and coworkers to assess whether the antihistamine azelastine could be used to treat COVID-19 [57]. Many proposed therapeutics are small molecules, but certainly not all of them, such as the work by Ratanabunyong et al. to assess the efficacy of an aptamer (short, single-stranded nucleic acid) inhibitor of human immunodeficiency virus type-1 reverse transcriptase (HIV-1 RT) [37]. Antibodies and antibody derivatives are also used as drugs, such as the artificial antibody-like peptide that Zheng et al. use to target the biomarker HER2 from the human epidermal growth factor receptor family [169]. Nanobodies are single-domain antibody fragments derived from camelids and have been explored by groups working to create potential therapeutics or inhibitors. One example is the work by Deng et al. to characterize a nanobody raised against proteins critical to the replication and amplification of chikungunya virus as a possible antiviral strategy [176].

SPR is also a popular technique to measure the kinetics of potential affinity reagents [5,24,30,34,35,36,39,82,94,96,106,110,111,120,132,138,141,177]. Lu and coworkers selected an aptamer to detect di(2-ethylhexyl) phthalate (DEHP), a plasticizer commonly used as an additive in food packaging with toxic effects as an androgen antagonist [24]. As with therapeutics, antibodies and antibody derivates are also popular agents to detect targets of interest, as in the work by Klangprapan and coworkers to develop a single-chain variable fragment (scFv) for the detection of porcine circovirus type 2 (PCV2) [141]. While many affinity reagents are developed for the purposes of detection, they are also a topic of interest to develop targeted therapies, such as the work by De La Fuente and coworkers to develop RNA aptamers for targeted chemotherapy to myeloid cells [111]. The ease of SPR, along with the ability to use it to characterize such a wide variety of potential analytes, makes it a popular technique to assess potential affinity reagents in the effort to develop better detection and better therapies.

While many studies use SPR to investigate one main binding pair (such as a target of interest and a potential therapeutic agent), SPR is also used to compare the binding kinetics of wild-type proteins to mutants [28,47,95,150,152], offering insights into how amino acid sequence and protein structure affect binding to the target of interest. Piazza et al. used the OpenSPR to study the binding of calmodulin (CaM), a Ca^2+^ control element in enzymes such as nitric oxide synthase (NOS) that are key in physiological processes, to the endothelial NOS peptide (eNOS). The OpenSPR was used to compare the binding of CaM to eNOS in buffers with different concentrations of Ca^2+^ in order to take into account the physiological concentration of Ca^2+^, which previous studies had not included [27]. Their next paper expands on the effects of Ca^2+^ on CaM binding to NOS peptides by creating mutants with substitutions of amino acids key to Ca^2+^ binding [28].

### 4.2. Applying Binding Kinetics: How OpenSPR Has Been Used to Study Disease States

The targets researchers have investigated with the OpenSPR vary widely. Many have focused on important challenges to human health. Several disease states emerge as common areas of study, with many researchers approaching the same problem with the same instrument in different ways. Three disease states frequently studied by OpenSPR users are Alzheimer’s disease [43,44,45,46], non-small cell lung cancer [47,48,49,50,51], and COVID-19 [34,52,53,54,55,56,57,58,59,60,61,62,63,64,65,66,67,68,69,70,71]. An overview of SPR research on these pathologies is included here, to demonstrate the versatility of the instrument on different kinds of biochemical systems, and to show some of the ways that researchers have used OpenSPR measurements of binding affinity and kinetics to solve critical research questions.

#### 4.2.1. Alzheimer’s Disease

Alzheimer’s disease is the leading cause of disabilities in the elderly and represents a growing concern in human health as some countries trend towards more aged populations [46]. One of the most characteristic phenomena leading to the development of AD is aberrant protein modifications leading to protein plaque formation by beta-amyloid_1–42_ (Aß) and tau proteins [43,44,45].

Aberrant post-translational modifications (PTMs) of tau protein such as hyper-phosphorylation, truncation/cleavage, and aggregation contribute to the progression of AD. Yang et al. aimed to understand how these PTMs are affected by the compound cornel iridoid glycoside (CIG), which is cited for both anti-inflammatory activity and improving memory. The OpenSPR was used to study the binding affinity between components of CIG and tau protein [44].

The chiral inversion of Aß due to a natural, spontaneously occurring PTM featuring a D-amino acid substitution is believed to be involved in the development of AD pathology. Work by Li and coworkers sought to understand the implications of this PTM on the role of Aß chiral chemistry and its role in plaque deposition. The SPR was used to compare the binding kinetics of full sequence Aß and chiral Aß C-terminal fragments to the tetrameric transthyretin Aß receptor, elucidating how exactly this PTM modifies binding behavior [43].

Human glutaminyl cyclase (hQC) is a zinc enzyme in the acyl transferase family and catalyzes the formation of pyroglutamate (pE)-modified Aß peptides. hQC is implicated in some pathologies, including AD, due to the catalysis of pE formation on N-terminally truncated Aß peptides. An accumulation of neurotoxic pE-Aß_3–40/42_ peptides in brain plaques is characteristic of the development of AD. Tsai et al. screened an array of potential hQC inhibitors from natural products and used the OpenSPR to characterize the most promising candidates [46].

While the role of Aß in AD is the focus of considerable study, poor sensitivity and selectivity of current Aß detection methods cause considerable obstacles to researchers. Nangare et al. aimed to address this difficulty by utilizing the OpenSPR to develop a graphene oxide-based biosensor with fg/mL sensitivity to detect the biomarker [45].

#### 4.2.2. Non-Small Cell Lung Cancer

OpenSPR has been applied to the study of many different types of cancer with a wide variety of molecular targets for each subtype studied in that effort. One critical cancer subtype explored by groups utilizing the instrument is NSCLC. Lung cancer is the leading cause of cancer-related deaths worldwide and NSCLC is the main histological subtype, accounting for 85% of all lung cancers [49,50]. Cases of NSCLC tend to be diagnosed at late stages when it is no longer possible to perform surgical resection. In addition, high rates of drug resistance and recurrence mean that five-year survival rates are extremely low, motivating efforts to improve detection methods and therapeutic strategies [47,50].

The signal transducer and activator of transcription 3 (STAT3) is an important target in many types of cancer intervention, including NSCLC, due to its activation in most human cancers. It regulates key processes in cancer such as cell proliferation, metastasis, and immune suppression, motivating the search to identify or develop inhibitors for STAT3. Shen and coworkers investigated telocinobufagin, a compound derived from traditional Chinese medicines that had reported antitumor effects. OpenSPR was used to determine the binding kinetics of telocinobufagin to STAT3 to explore the possibility of using telocinobufagin as a therapeutic STAT3 inhibitor [50].

Some NSCLC patients with driver oncogenes for epidermal growth factor receptor (EGFR) mutations can be successfully treated via targeted therapies [50]. Zhang et al. assessed the potential of almonertinib, a new EGFR tyrosine kinase inhibitor (TKI) that may be more effective for patients whose cancer has metastasized. Almonertinib has been proposed as a better option than osimertinib, a medication with demonstrated efficacy but detrimental side effects. They used OpenSPR to compare the binding affinity for both almonertinib and osimertinib to EGFR mutant protein as part of their characterization [49].

Tumor-necrosis-factor-related apoptosis-inducing ligand (TRAIL) was developed as an anticancer therapy but failed as a late-stage treatment due to TRAIL resistance in many NSCLC tumors. Zhang and coworkers identified the compound curcumol as a sensitizer that can overcome TRAIL resistance in NSCLC while screening a food-source compound library. They identified NRH:quinone oxidoreductase 2 (NQO2) as the molecular target of curcumol, and used the OpenSPR to compare binding between curcumol and NQO2 with curcumol to aNQO2 mutant to better understand the mechanism of binding and which amino acid residues were responsible for interacting with the inhibitor [47].

The interaction between ß-catenin and lymphoid enhancer factor 1 (LEF1) has been identified as a key therapeutic target of anticancer therapies due to the role of the Wnt/ß-catenin signaling pathway’s role in metastasis. Chen et al. developed a high-throughput screening assay to identify possible inhibitors of this interaction. They used OpenSPR to test the most promising candidates for binding affinity to ß-catenin [48].

Kinases have been suggested as possible biomarkers for some diseases due to the role they play in important cellular mechanisms. Solomon et al. developed a biosensor for the ERK2 kinase utilizing a peptide derived from hepatoma-derived growth factor (HDGF) protein. ERK2 plays a role in activating HDGF, and overexpression of the protein is a biomarker for various diseases, including NSCLC. SPR was used to characterize the binding kinetics of ERK2 and a HDGF-derived peptide in order to assess the usefulness in developing a biosensor based on a peptide monolayer of HDGF [51].

#### 4.2.3. COVID-19

OpenSPR emerged as a useful tool in the race to develop diagnostics and therapeutics to treat COVID-19. In September of 2020, the first paper citing the use of Nicoya OpenSPR on applications to study COVID-19 appeared [53]. Understandably, many of the papers (particularly early in the pandemic) focus on finding or developing potential treatments for COVID-19. The vast majority of papers exploring this topic were predicated on the cited role of ACE2 protein in allowing the virus to enter host cells and testing both new and old compounds to treat the disease by blocking host-cell interactions with ACE2 [52,53,61].

Eleven groups looked at potential small molecules to inhibit ACE2 binding, and SPR was used to supplement that goal by assessing binding affinity and kinetics of the potential therapeutic small molecules with ACE2 protein [52,53,56,57,58,59,60,61,62,66,67]. Three groups approached the goal of potential therapeutics from a different direction: they chose targets for inhibition on SARS-CoV-2 itself, rather than starting with host ACE2 protein. Du and coworkers investigated the 3CLpro protease [55], and both Singh et al. and He et al. looked at SARS-CoV-2 spike proteins [65,71].

In addition to measuring the binding of small molecules puerarin and quercetin to ACE2 to confirm binding, the work by Pan et al. goes a step further with the use of SPR and included competition assays to study the effect of their inhibitors on viral S-protein binding to the ACE2 receptor [52].

The OpenSPR proved useful in the efforts to develop therapeutics, but the instrument was also used in studies assessing the structure and function of the virus and the mechanisms of host cell entry that affect pathogenicity [54,64,70], in studies developing potential detection methods to control the spread of COVID-19 [63,68,69], and in one study that was dedicated specifically to developing an LSPR aptasensor platform [34].

## 5. Using the OpenSPR to Benchmark and Compare to Other Methods

The OpenSPR is often used to supplement investigations into discovering potential inhibitors or finding affinity reagents in studies with many different experimental techniques, but perhaps the best way to highlight how the OpenSPR has been used in tandem with other methods is to look at cases when the instrument has been used as a direct comparison to other experimental methods or in silico docking calculations.

The troponin protein complex is critical to the regulation of striated muscle contraction and relaxation, but traditional methods to study the structure–function relationships within the complex require large amounts of purified protein or protein labeling that may change conformation. Rasmussen and Jin approached this challenge by developing site-specific monoclonal antibody probes to use in a high-throughput ELISA assay. The SPR offers kinetic information about binding partners of interest while ELISA is an endpoint measurement of affinity, and they used the SPR to validate the results of their microplate assay [196]. Deshmukh et al. wanted to investigate the effects of truncations to streptococcal enolase (from Streptococcus pyogenes) on its ability to bind to canine plasminogen. Their primary methodology was dual polarization interferometry (DPI), but they wanted to validate that the results were independent of the type of immobilization used. For this reason, they used the OpenSPR with both gold and Ni-NTA sensor chips [93]. Studies by Serrano and coworkers into the disassembly of cholera and *Escherichia coli* (*E. coli*) holotoxins were expanding on work previously carried out via ELISA using hybrid versions of the toxins. They demonstrated differences when the assay was performed on the native forms of the toxins and validated their results with SPR. Both methods demonstrated the same trend for the analytes, and the researchers attribute the difference in how complete the disassembly was between the assays to the set-up of the techniques, given that the SPR has shearing forces from the running buffer whereas the plate-based ELISA assay does not [214].

In addition to using the SPR to benchmark conventional experimental techniques, some groups used the SPR to verify in silico calculations. Hao et al. used molecular docking scores to screen potential affinity peptides to detect the porcine circovirus type II (PCV2) capsid protein. This was followed by using the OpenSPR and ELISA to characterize the peptides experimentally [96]. Similarly, the work by Yu and coworkers comparing two methods of calculating protein docking was also verified with ELISA and SPR [90].

## 6. Beyond Simple Kinetics: The Versatility of OpenSPR

SPR is an extremely useful and versatile technique. In addition to the oft-cited use of the OpenSPR instrument to measure binding kinetics of ligand–analyte pairs, the SPR can be used to measure a variety of other phenomenon, including ligand aggregation and aggregation-based inhibition [213], assembly and disassembly of holotoxin complexes [214,215], drug release [216], collaborative binding [211,212], competition studies [217,218], and inhibition assays [15,219]. SPR has also been used to characterize the immobilization of an affinity reagent, rather than testing binding to the target itself. Churcher, Upasham, and coworkers utilized SPR for the real-time monitoring of antibodies onto a Protein A functionalized surface, to verify they could attach the molecule of interest to a biosensor that relied on a different method of detection [220,221,222]. While many papers use SPR to supplement other investigations, some studies focused on developing biosensors based entirely on the technique of LSPR itself [34,45].

### 6.1. Aggregation

Though most SPR experiments require the immobilization of a specific binding partner of interest, the real-time kinetic measurement technique can be extremely useful for measuring non-specific ligand aggregation. Typically, SPR experiments are designed to avoid non-specific, but a study by Boulton et al. focused on the problem of ligand self-association that can cause false positives in the process of attempted drug discovery [213]. They used a nonfunctionalized gold surface and demonstrated the ability to detect aggregation of their analyte of interest, ESI-09, at a concentration above the critical aggregation concentration (CAC). Human serum albumin (HSA) is a common additive to attenuate problems of self-aggregation. Once they established the CAC, they used SPR to determine whether HAS could prevent the nonspecific aggregation of ESI-09 above the CAC. While the OpenSPR is not suitable for studying all types of aggregates, it can be a powerful tool for rapidly testing experimental conditions to alleviate common pitfalls (such as self-aggregation) to improve the process of drug discovery [213].

### 6.2. Disassembly

SPR is most often used to measure binding, but it can be used to measure disassembly as well. Huhn et al. utilize the OpenSPR to study the disassembly of a cytolethal-distending toxin (CDT) holotoxin. The protein is composed of three domains, designated CdtA, CdtB, and CdtC. The complex was immobilized on the surface using an antibody and the baseline was set to zero before measuring the drop in signal when the buffer pH was changed. Probing the system with antibodies specific to the individual domains allowed researchers to determine which domains were lost at each respective pH condition [215].

In a similar experiment, Serrano et al. studied the differences between cholera toxin (CT) and *E. coli* heat-labile enterotoxin (LT) using the OpenSPR. Protein disulfide isomerase (PDI) can separate the subunits of both toxins, but this study demonstrates that structural differences between CT and LT result in a marked difference in how efficiently they are disassembled by PDI, which has implications for toxicity [214].

### 6.3. Tuning Drug Release

Luo et al. created a tunable drug-delivery system via hybrid nanofibers. Graphene oxide was used because it is non-degradable and thus retains the payload of interest. The researchers combined graphene oxide nanofibers with manganese dioxide, which is biodegradable and thus enabled drug release in the cell. By tuning the ratio of the two, they could optimize drug delivery and release. They established a nanomaterial layer on amine-functionalized chips and then captured BSA onto the platform. They then characterized biodegradability using ascorbic acid to release the captured BSA, using the drop in signal to monitor the time for BSA release by the nanomaterial sensor [216].

### 6.4. Collaborative Binding

By immobilizing one of a dual regulator pair on their sensor surface and then measuring interaction with either the coregulator or DNA-containing binding sequences as opposed to both at once, Heacock-Kang et al. demonstrated that both of their regulators of interest, PA1413 and PA1226, are required for binding to DNA in *Pseudomonas aeruginosa* [212]. The same technique was used by the group to study a separate pair of regulators, PA3898 and PA2100 [211].

### 6.5. Competition Studies

Work by Zheng et al. focused on developing a vaccine for overdose protection against ketamine used the OpenSPR to perform competition studies of BSA-hapten cognates in the presence of increasing concentrations of ketamine (or its metabolites) to assess the utility of their potential vaccine [218].

Many studies on developing therapeutics focus on proposing potential inhibitors to targets of interest. A study by Suetaka and coworkers focuses on the interaction between transcription factor c-Myb and the kinase-inducible domain (KIX) because aberrant expression of c-Myb is associated with leukemia. They designed mutants of the c-Myb transactivation domain (TAD) and tested the binding of c-Myb to KIX in the presence of said inhibitor to demonstrate the efficacy of their inhibitor [219].

### 6.6. Endogeneous Binding

SPR is typically carried out on purified binding partners; in fact, many proteins are purified via protein tag such as 6xHis, and that same tag can be used to immobilize the ligand to the SPR sensor. However, Li et al. paired the process of sample separation with their method of affinity detection using the OpenSPR. Bcl-xL (an anti-apoptotic member of the B cell lymphoma 2 family) is a key factor of interest in tumorigenesis, but the regulation by the microenvironment is critical to understanding binding. Their other binding partner of interest was the ligase RING finger protein 152 (RNF152). They immobilized a Bcl-xL antibody onto a carboxyl sensor chip and used it to capture bound Bcl-xL-RNF152 complex from cell lysate. They then utilized an RNF152 antibody to determine the binding of Bcl-xL and RNF152 within the cell. Using the SPR to separate out binding partners of interest (instead of introducing binding between two purified samples, as most experiments do) simplifies sample handling and offers insight into binding that occurred within the cell, in order to more closely approximate the conditions of interest [223].

### 6.7. Custom Sensors

The vast majority of studies utilize the commercially available functionalized sensor chips available from Nicoya (Table 3). However, a few groups functionalized the sensors themselves or had custom applications.

Krueger and coworkers functionalized standard gold sensor chips with 12-mercaptododecanoic acid followed by NHS/EDC crosslinking of peptides [29]. In the work by Wang et al. focused on developing an LSPR sensor for enrofloxacin, they used polydopamine molecular-imprinted polymer functionalized in-house as the detection element [160].

While some groups started with standard gold sensor chips from Nicoya and performed their own surface functionalization as above, Cathcart et al. utilized the instrument in a less traditional manner. They used a modified OpenSPR instrument with a cuvette in place of a flow cell to monitor the synthesis of custom pentagonal silver star nanoparticles [72]. The same group also explored metal oxide encapsulated nanoparticles and shell rebuilding of silver nanoparticles to tune LSPR resonance wavelength, again utilizing an OpenSPR to characterize their nanoparticles [209,210].

## 7. Conclusions

In the decade since Nicoya’s founding, their first instrument has seen an impressive reach, aiding in research ranging from developing therapeutics for cancer and other diseases to characterizing nanoparticles that may one day be used to create new kinds of SPR sensors. The first three papers citing the use of the OpenSPR appeared in 2016, and the instrument has seen rapid growth in the years since, with 68 papers citing the use of the OpenSPR in 2021 and 50 in 2022. SPR is a rapid and useful technique to study biochemical systems of interest in a wide variety of potential applications, and the OpenSPR will likely remain a widely used tool for investigators that are interested in one set of binding partners or a small handful, as with most of the papers cited in this review. However, in the past few years, Nicoya has debuted the Alto, a new high-throughput SPR platform that leverages the use of digital microfluidics for automatic sample dilutions and sensitive measurements with extremely low sample requirements. The Alto combines many of the benefits of the OpenSPR with simplified sample handling, but it also introduces new experimental capabilities that would have been immensely labor-intensive or not possible on the OpenSPR such as epitope binning or library screening [76,224]. It will be exciting to see what the next decade brings for benchtop SPR instruments.

## Figures and Tables

**Table 1:** Number of papers citing the use of a Nicoya OpenSPR by year.

Year	Number of Papers
2016	3
2017	4
2018	20
2019	20
2020	35
2021	68
2022	50

**Table 2:** Parameters of the Nicoya OpenSPR [74] when compared with the company’s new high-throughput SPR system Alto [75,76] and similar commercial instruments from Cytiva (Buckinghamshire, UK) [77] and Bruker (Billerica, MA, USA) [78,79]. * The baseline drift and baseline noise numbers for the OpenSPR were taken from a representative experiment carried out by our lab.

Parameter	OpenSPR-XT (Nicoya)	Alto (Nicoya)	Biacore X100 (Cytiva)	Sierra SPR-32 Pro (Bruker)
Association Rate (1/M·s)	1 × 10^3^–1 × 10^7^	up to 1 × 10^9^	1 × 10^3^–1 × 10^8^	1 × 10^3^–1 × 10^7^
Dissociation Rate (1/s)	1 × 10^−5^–0.1	1 × 10^−5^–1.0	1 × 10^−5^–0.1	1 × 10^−6^–0.1
Affinity Range	pM–mM	pM–mM	pM–mM	pM–mM
Channels	2	16	2	8
Injection	100 μL	-	5–90 μL	2–200 μL
Sample Volume	200 μL	2 μL	Injection + 20–30 μL	Injection + 10–35 μL
Flow Rate	5–200 μL/min	-	1–100 μL/min	5–100 μL/min
Sample Capacity	Two 96-well plates	48 analytes	15 vials	Two microwell plates (96- or 384-well)
Temperature	4–40 °C	Off, 25, 37 °C	Ambient (4–40 °C with Plus Package)	10–37 °C
Baseline Drift	~1.2 RU *	-	<0.3 RU/min	<0.15 RU/min
Baseline Noise	~0.44 RU (RMS) *	-	<0.1 RU (RMS)	0.02 RU (RMS)
Automated Run Time	24 h	24+ h	24 h	unlimited

**Table 3:** Nicoya OpenSPR Commercial Sensor Chips Cited in Scientific Literature.

Sensor Type	Number of Studies Utilizing Chip
Carboxyl	118
NTA	48
Streptavidin	22
Gold	7
Unknown	6
GST	2
Hydrophobic	2
Liposome	1

## Data Availability

Data sharing not applicable.
